# Tomato Juice Supplementation Influences the Gene Expression Related to Steatosis in Rats

**DOI:** 10.3390/nu10091215

**Published:** 2018-09-02

**Authors:** Laura Inés Elvira-Torales, Inmaculada Navarro-González, Rocío González-Barrio, Gala Martín-Pozuelo, Guillermo Doménech, Juan Seva, Javier García-Alonso, María Jesús Periago-Castón

**Affiliations:** 1 Department of Food Technology, Food Science and Nutrition, Faculty of Veterinary Sciences, Regional Campus of International Excellence “Campus Mare Nostrum”, Biomedical Research Institute of Murcia (IMIB-Arrixaca-UMU), University Clinical Hospital “Virgen de la Arrixaca”, University of Murcia, Espinardo, 30071 Murcia, Spain; inmaculada.navarro@um.es (I.N.-G.); rgbarrio@um.es (R.G.-B.); galamartin@um.es (G.M.-P.); gdomenech@um.es (G.D.); fjgarcia@um.es (J.G.-A.); 2 Department of Food Engineering, Tierra Blanca Superior Technological Institute, Tierra Blanca, 95180 Veracruz, Mexico; 3 Department of Histology and Pathologic Anatomy, Faculty of Veterinary Sciences, Regional Campus of International Excellence “Campus Mare Nostrum”, University of Murcia, Espinardo, 30071 Murcia, Spain; jseva@um.es

**Keywords:** lycopene, non-alcoholic fatty liver disease (NAFLD), oxidative biomarkers, gene expression, quantitative metabolomics

## Abstract

The objective of this work was to identify the effect of tomato juice on the expression of genes and levels of metabolites related to steatosis in rats. Male Sprague Dawley rats (8 weeks-old) were grouped (6 rats/group) in four experimental groups: NA (normal diet and water), NL (normal diet and tomato juice), HA (high-fat diet and water), and HL (high-fat diet and tomato juice). After an intervention period of 5 weeks, rats were sacrificed and biochemical parameters, biomarkers of oxidative stress, liver metabolites, and gene expression were determined. Although the H diet provoked dislipemia related to steatosis, no changes in isoprostanes or liver malondialdehyde (MDA) were observed. Changes in the gene expression of the HA group were produced by the high consumption of fat, whereas the consumption of tomato juice had different effects, depending on the diet. In the NL group, the genes involved in β-oxidation were upregulated, and in groups NL and HL upregulation of *CD36* and downregulation of *APOB* and *LPL* were observed. In addition, in the HL group the accumulation of lycopene upregulated the genes *FXR* and *HNF4A*, which have been suggested as preventive factors in relation to steatosis. Regarding the metabolomics study, intake of tomato juice stimulated the biosynthesis of glutathione and amino acids of the transulfurization pathway, increasing the levels of metabolites related to the antioxidant response.

## 1. Introduction

Non-alcoholic fatty liver disease (NAFLD) affects both developed and developing countries and is forecast to reach pandemic proportions [1]. Due to the increased incidence of obesity worldwide, NAFLD has become an important public health problem because of its high prevalence, potential progression to severe liver disease, and strong link with important cardiometabolic risk factors [2]. NAFLD is a group of liver diseases ranging from hepatic steatosis to non-alcoholic steatohepatitis, and progressing to fibrosis, cirrhosis, liver failure, hepatocellular carcinoma, or death [3]. It is the hepatic manifestation of the complex metabolic derangements associated with obesity. NAFLD is characterized by excessive deposition of fat in the liver (steatosis) and develops when hepatic fatty acid availability from plasma and de novo synthesis together exceed hepatic fatty acid disposal by oxidation and triglyceride export. Hepatic steatosis is, therefore, the biochemical result of an imbalance between complex pathways of lipid metabolism, and is associated with an array of adverse changes in glucose, fatty acid, and lipoprotein metabolism across all tissues of the body [4].

The Mediterranean diet has been proposed as an appropriate alternative for the reduction and maintenance of body weight and to reduce steatosis, and liver inflammation and fibrosis. For this reason, it has been recommended for the treatment of NAFLD in the practical clinical guide of the European associations for the study of the liver, diabetes, and obesity (EASL-EASD-EASO) [5]. The Mediterranean diet combines marine products and grains rich in mono and polyunsaturated fats with fruits and vegetables rich in dietary bioactive antioxidants, such as polyphenols and carotenoids, which have a beneficial effect in the prevention of NAFLD, as has been shown in different animal models [1,6,7,8]. Interestingly, for non-provitamin A carotenoids, and particularly for lycopene (from tomato) [3,9], there is much experimental evidence on the prevention of steatosis and inflammation in murine models, through different mechanisms, including the scavenging activity of reactive oxygen species. In a previous study, we reported that the consumption of tomato juice improves the lipid metabolism in rats with steatosis induced by a fatty diet and acts as a regulator of lipid metabolism improving β-oxidation [4,10]. Moreover, lycopene exerts efficient anti-inflammatory and antiproliferative effects, which also act against NAFLD [3,7,11]. The intake of tomato juice by individuals on a hypercholesterolemic and high-fat diet partially recovered amino acid levels in liver, particularly for L-carnitine, which could indicate a better ability to maintain the mitochondrial activity, concerning β-oxidation, and to protect the fatty liver against inflammation and fibrosis [10]. These metabolic changes were accompanied by an overexpression of genes involved in the mitochondrial and peroxisomal β-oxidation, contributing to more efficient oxidation of long-chain fatty acids [10]. These effects can be due to direct interaction of carotenoids molecules or their derivatives with nuclear transcription factors (PPAR; RXR, RAR, LXR) and/or indirect modification of transcriptional activity through changes in the cellular redox status [12]. Particularly, PPARs play an important role in the regulation of fatty acid oxidation and lipoprotein metabolism, and in the inflammatory and vascular response [13]. Other authors have proposed that lycopene can exert this effect via micro-RNA modulation. These are small, non-coding RNAs that are involved in post-transcriptional regulation of target genes, regulating lipid synthesis, fatty acid oxidation, cholesterol metabolism, and lipoprotein formation and secretion [6].

Because tomato juice is a natural source of lycopene, utilizing a whole-food intervention approach provides multiple nutrients with a broad range of biological activities, creating the potential for complementary, additive, or synergistic activities that are lacking when supplementation involves only a single nutrient. In this sense, tomato juice intake partially ameliorated high-fat-induced disturbances, particularly by increasing *Lactobacillus* abundance and diminishing the acetate:propionate ratio, suggesting a potential improvement of the metabolic aspects of NAFLD, mainly due to the presence of non-digestible carbohydrates [14].

These findings could lead to new targets for therapeutic agents against NAFLD; hence, it is important to elucidate the metabolic and genomic mechanisms influenced by lycopene and to achieve a better understanding of the role of lycopene in liver metabolism. Therefore, the objective of the present study was to identify the effect of tomato juice on the expression of genes related to steatosis in rats induced by diet, accompanied by monitoring of the metabolites involved in this condition, with the purpose of understanding the mechanisms that act in the control of NAFLD.

## 2. Materials and Methods

### 2.1. Tomato Juice

Tomato juice was obtained from a local producer and the total content of bioactive compounds was analyzed according to previously published methods [15,16]. The nutritional composition of this juice was: protein 0.8%, fat 0.1%, natural sugars 4.7%, dietary fiber 0.8%, and lycopene 12 mg/100 mL.

### 2.2. Animals and Experimental Design

The experiment was carried out following the guidelines approved by the Bioethics Committee of the University of Murcia. In brief, 24 male Sprague-Dawley rats, 8 weeks-old were purchased in Charles River Laboratories España S.A. Rats were received in the Animal Research Centre of Murcia University, and were maintained under controlled temperature (22 °C), air humidity (55%) and 12-h light–dark cycle conditions for 2 weeks before starting the experiment. During this period, they had free access to diet and tap water. During this period, they were randomly divided into two groups (*n* = 12), one of which was fed a standard laboratory diet (Teklad Global 14% Protein Rodent Maintenance diet, 4.0% fat Harland Laboratories, Indianapolis, IN, USA) and the other a hypercholesterolemic and high-fat diet (Atherogenic rodent diet TD-02028, with 21.2% fat, 1.25% cholesterol, and 0.5% cholic acid, Harland Laboratories, Indianapolis, IN, USA).

Animals from N and H groups were randomly divided into two other groups, giving four experimental groups (*n* = 6), according to the diet (N and H) and drink (tomato juice, as a natural source of lycopene, or water): NA (normal diet and water), NL (normal diet and tomato juice), HA (hypercholesterolemic diet and water) and HL (hypercholesterolemic diet and tomato juice). After an intervention period of 5 weeks, rats were euthanized and blood and liver samples were obtained, which were stored at −80 °C until analysis.

### 2.3. Histopathological Examination

The samples were prepared in paraffin blocks and cross-sections (4 μm thick) were stained with hematoxylin and eosin (H-E) for examination with a light microscope. Steatosis was evaluated as described by Brunt et al. [17]

### 2.4. Biochemical Parameters of Plasma

The levels of glucose, total protein, total cholesterol, high-density lipoprotein (HDL) and low-density lipoprotein (LDL) cholesterol, and total triglycerides (TGA) and the activities of the enzymes alanine and aspartate aminotransferase (ALT and AST) were analyzed using an automatic analyzer (AU 600 Olympus Life, Hamburg, Germany). The very low-density lipoprotein (VLDL) cholesterol was estimated according to Friedewald et al. [18]

### 2.5. Determination of Biomarkers of Oxidative Stress

The content of urinary 15-F_2t_-isoprostane (8-epi-PGF_2α_) was determined with an enzyme-linked immunosorbent assay (ELISA) kit (OxiSelectTm 8-*epi*-PGF_2α_ Elisa Kit, Cell Biolabs, San Diego, CA, USA); it was normalized by measurement of the creatinine concentration [19]. Lipid peroxidation was analyzed as the malondialdehyde (MDA) level in the liver, measured with the high-performance liquid chromatography (HPLC) method described by Mateos et al. [20]. The liver protein concentration was determined by Lowry’s method, as modified by Bensadoun and Weinstein [21].

### 2.6. Analysis of Lycopene by High-Performance Liquid Chromatography (HPLC)

The analysis of lycopene and its metabolites was adapted from Seybold et al. [22]. The liver samples were thawed and 0.5 g were extracted three times with methanol/tetrahydrofuran (1/1, *v*/*v*) containing 0.1% butyl hydroxytoluene. The extracts were combined and dried under vacuum at 37 °C. The residues were suspended in 2 mL of ethanol, centrifuged at 20,817 g for 10 min at room temperature, filtered, and analyzed by LC/MS using an Agilent (Waldbronn, Germany) MSD single quadrupole mass spectrometer, using the conditions described by Martín-Pozuelo et al. [10] for the determination of (*E*) -lycopene, (*Z*) -lycopene, and the 6-, 8-, and 12-apo-lycopenals.

### 2.7. Study of the Expression of Genes Involved in Fatty Liver Disease

Total RNA was extracted from frozen liver using a TNeasy^®^ Mini Kit (Qiagen, Duesseldorf, Germany). We used a 96-well PCR array designed for the evaluation of fatty liver disease genes (PARN-157ZD-24, Qiagen, SABiosciences, Frederick, MD, USA). This array includes genes for insulin signaling, adipokines, the inflammatory response, apoptosis, and carbohydrate and lipid metabolism in the liver. The genes of the rats in the different treatment groups were analyzed using the RT^2^ Profiler^TM^ PCR Array Data Analysis. The gene transcription results were considered significant if the change, at the *p* < 0.05 confidence level, was ≥1.5-fold or ≤−1.5-fold.

### 2.8. Analysis of Liver Metabolites by HPLC-Mass Spectrometry (MS)

The extraction and analysis of metabolites were carried out as described by Bernal et al. [6]. The analysis was carried out in an HPLC instrument (1200 series; Agilent Technologies, Santa Clara, California, USA) coupled to an Agilent 6120 single quadrupole mass spectrometer with an orthogonal ESI source. It is advisable to perform the extraction process and the analysis in a single step, since freezing or lyophilization degrades the sample and there may be an alteration of the results [6,23].

### 2.9. Statistical Analysis

To perform the statistical analysis of the analytical parameters in the four experimental groups, a one-way analysis of variance (ANOVA) was carried out together with a Tukey post-hoc test. In addition, for the parameters with initial and final values and in the analysis of the lycopene content, the values were compared using a paired *t*-test. For the analysis of liver metabolites, the concentrations were normalized according to the weight of the tissues, and for statistical analysis a two-way ANOVA was carried out followed by a *t*-test; its familywise error rate was corrected using the Benjamini–Hochberg false discovery rate (FDR) with a 5% proportion of false discovery [24]. The significance level was *p* < 0.05. The statistical analyses were performed with Minitab version 17.0; for the correction by FDR, the IBM Statistical Package for the Social Sciences (SPSS) version 19.0 was used.

## 3. Results

### 3.1. Weight Gain, Volume of Feed Consumed, and Excreta

Table 1 shows the initial and final weights of the rats, the consumption of food and drink, and the consumption and absorption of lycopene by the animals. No significant differences were observed in the final weight, and the body weight gain was 25.6% and 20.1% for HA and HL, respectively, and 15.4% and 14.7% for NA and NL, respectively, the values being greater in the H groups, as expected. A significant increase in food intake was found in the N groups with respect to the H groups; consequently, the same pattern occurred for the excreted feces, due to the reduced food intake of the H groups as a result of the high caloric value of the H diet. Regarding the intake of drinks, the L groups showed higher consumption (63.6 and 78.8 mL for NL and HL, respectively), the animals showing a preference for tomato juice, which resulted in significantly higher excretion of urine. For the mean values of lycopene intake and lycopene excretion in feces, no significant differences were observed, with an apparent absorption of 68.1% in HL rats and 55.7% in NL rats.

### 3.2. Contents of Lycopene and Its Isomers in the Liver

The contents of lycopene and its isomers and metabolites in the liver are shown in Table 2. The total contents of lycopene and its *trans* isomer were significantly higher in group HL, vs. NL. An average concentration of 1.05 μg/g of total *cis*-lycopene was recorded in both groups (the sum of 9-*cis* and 13-*cis* lycopene). However, the apo-lycopene metabolites were not detected in any sample.

### 3.3. Histopathological Examination and Biochemical Parameters

**Figure 1:**
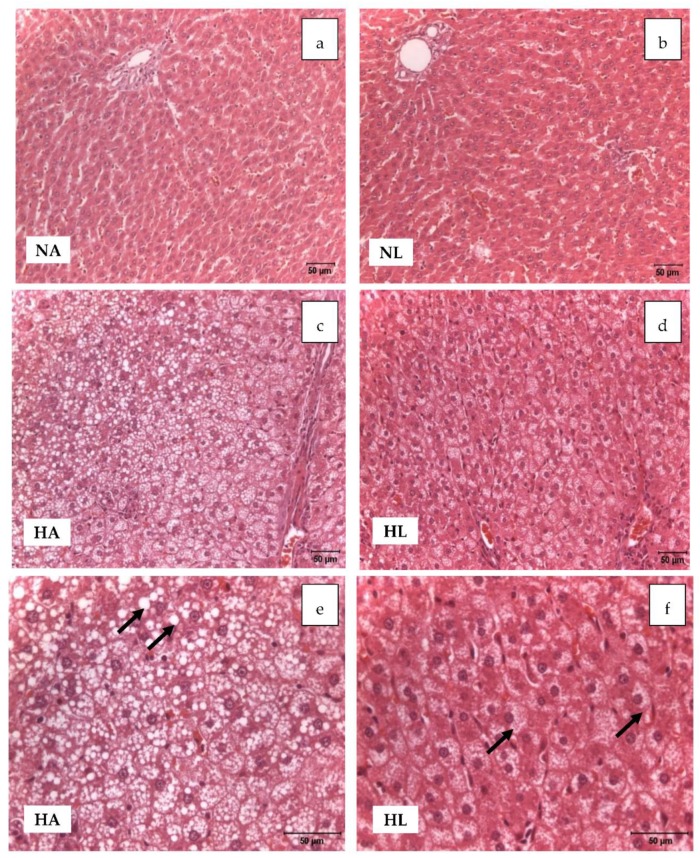
Light microscopic study of liver samples of animals of the four experimental groups (H–E x20; pictures (**a**–**d**), and H–E x40; pictures (**e**,**f**)). Pictures (**a**) and (**b**) show a normal liver structure. Pictures (**c**), (**d**), (**e**), and (**f**) show vacuolar degeneration of hepatocytes in groups HA and HL, due to the accumulation of fat. In HA, large vacuolar formations can be identified in some areas, whereas in HL there is a distribution of small droplets of fat.

Due to the high-fat diet, the HA and HL groups developed steatosis and hepatocyte ballooning, but to different degrees [13] since for the rats that drank tomato juice the lipid droplets were smaller (Figure 1e,f). Moreover, a mildly inflammatory infiltrate, composed of mononuclear cells, was detected in both groups due to the high content of fat (Figure 1). Additionally, elevated levels of hepatic aminotransferase enzymes (ALT and AST, Table 3) and a significant increase in the liver weight (Table 2) were found in the H groups at the end of the experiment; these values exceeded those of the NA group.

Nevertheless, it is notable that the serum levels of the enzymes were within the range of the reference values reported in the literature [25]. By contrast, livers from the rats of the N groups showed normal architecture and no evidence of steatosis.

Table 3 shows the results for the biochemical parameters of plasma. No differences among the groups were observed in the initial values (data not shown); the most important changes were detected in the final values, when comparing the H and N groups. Significantly higher levels of total cholesterol, LDL-cholesterol, VLDL-cholesterol, and TGA were detected in HA and HL rats at the end of the study, due to the dyslipidemia induced by steatosis. However, the HDL-cholesterol remained unchanged along the study. Regarding the biomarkers of oxidative stress (Table 3), significant differences between groups H and N were observed for the final concentrations of urinary isoprostanes. For MDA in liver the same behavior was observed, with significantly higher levels in the H groups; up to 4-times higher than in the N groups (Table 4). Tomato juice consumption and the accumulation of lycopene did not reduce the abundance of the oxidation biomarkers.

### 3.4. Expression of Genes in the Rat Liver

Table 5 shows the differentially expressed genes (*p <* 0.05) with a fold change greater than ±1.5. The genes with a significant fold change were related to fatty acid β-oxidation, and to the transport and metabolism of cholesterol and other lipids. The expression relative to the NA rats shows that both diet and tomato juice consumption modified the liver transcriptome. Ten, 15, and six genes were modulated in NL, HA, and HL rats, respectively, in comparison with the NA group. In the group supplied with tomato juice in the normal diet (NL), the genes involved in the β-oxidation, transport, and synthesis of fatty acids and cholesterol—*ACOX1, CD36, CYP7A1, SREBF2*, and *SCD1*—were overexpressed, but *LPL, ACSL5*, and *APOB* were down-expressed. It is of note that *NR1H2* and *IL10* also showed a significant increase in their relative expression, in comparison with that in NA rats.

In the HA group there was significant overexpression of the lipid transporter genes—such as *CPT2, CPT1A, ABCA1*, and *ABCG1*—but *CD36* was down-expressed. The genes related to the formation of lipoproteins, *APOB* and *LPL*, were also overexpressed. Regarding the metabolism of different lipids, *ACSL5, HMGCR*, and *DGAT2*, as well as *CYP7A1* (involved in the synthesis of bile acids), were upregulated. Moreover, the leptin receptor (*LEPR*) also showed significant overexpression. These alterations in gene expression are associated with the intake of the high-fat diet, and tomato juice consumption appears to have had a significant impact on the gene expression, because in HL rats significant upregulation was observed *for CD36, FABP5, PPA1*, and *HNF4A*, whereas *LPL* and *APOB* showed repression. Moreover, for HL, only *NR1H4* (nuclear receptor 1H4) and *HNF4A* (hepatocyte nuclear factor 4α) were significantly overexpressed, in comparison with HA.

### 3.5. Intracellular Levels of Liver Metabolites

**Figure 2:**
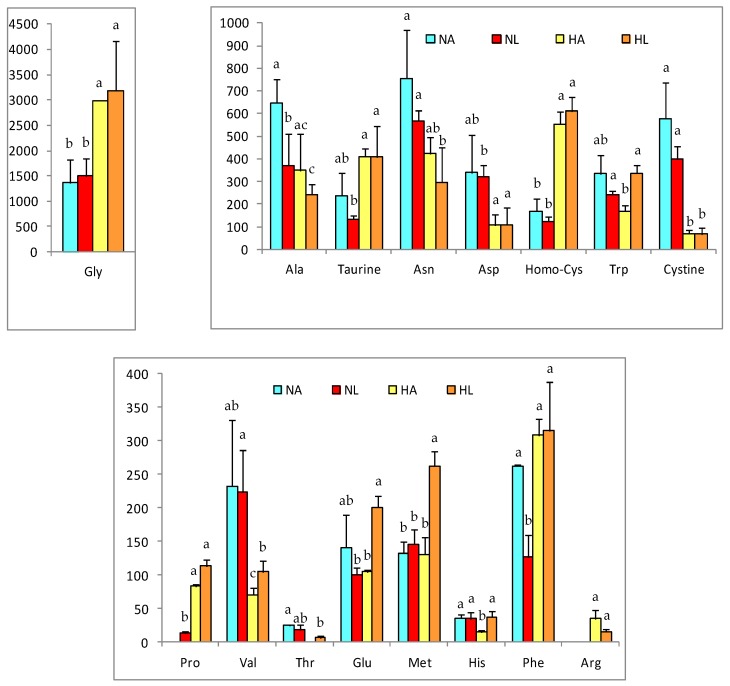
Relevant amino acids concentrations in liver of rats of the four experimental groups (NA: normal diet and water, NL: normal diet and tomato juice as drink, HA: hypercholesterolemic and high-fat diet and water, HL: hypercholesterolemic and high-fat diet and tomato juice). Concentration values are given in nmol/g, as mean ± SD. ^a–c^ Different letters indicate a statistically significant difference (*p* < 0.05).

Although we did not determine the proteins encoded by the differentially expressed genes, we did analyze the metabolites involved in several metabolic functions. The influence of the diet and the intake of tomato on these amino acids is shown in Figure 2.

The high-fat diet induced greater metabolic changes; however, the consumption of tomato produced changes in the antioxidant profile. For most of the metabolites analyzed, their concentration in the liver was decreased by the fatty diet, except for glycine, proline, taurine, phenylalanine, and homo-cysteine; nonetheless, arginine- not detected in the N groups- had a mean value of 34.9 nmol/g in HA and 15.9 nmol/g in HL. Important changes were also detected in the liver concentrations of methionine, histidine, and glutamic acid, which were all increased in HL rats.

**Figure 3:**
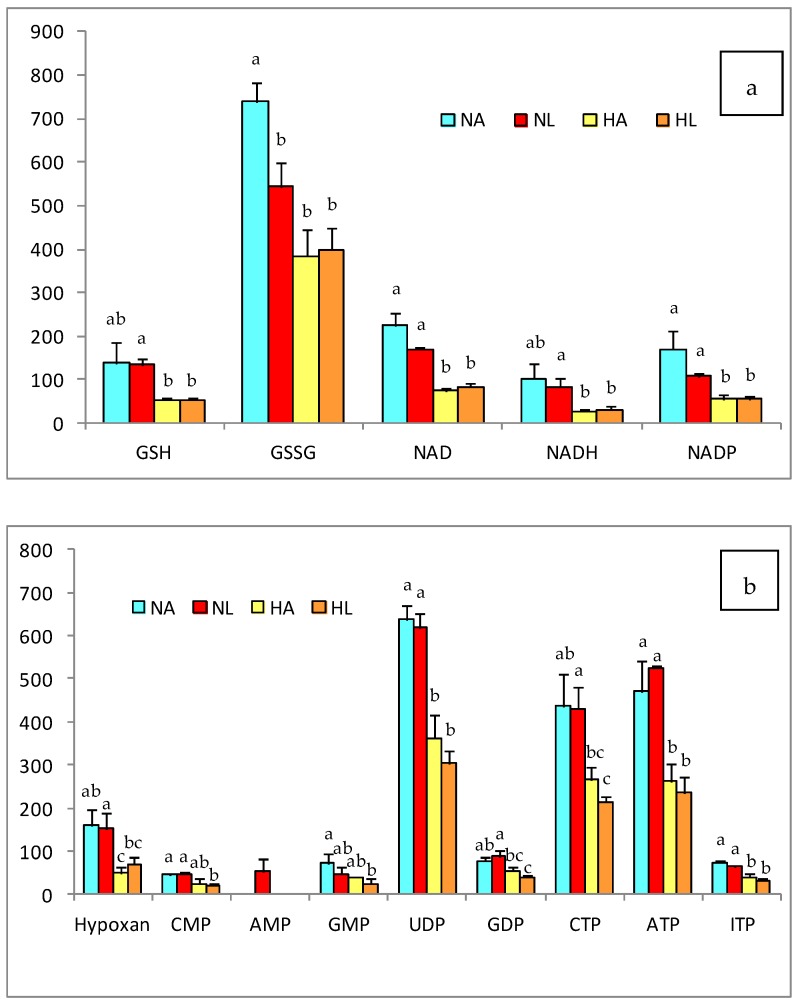
(**a**) Antioxidant and (**b**) nuleotide compounds in the liver of rats of the four experimental groups (NA: normal diet and water, NL: normal diet and tomato juice as drink, HA: hypercholesterolemic and high-fat diet and water, HL: hypercholesterolemic and high-fat diet and tomato juice). The bar height indicates the mean value for each group and the error bar indicates the standard deviation. ^a–c^ Diferent letters indicate a statistically significant difference (*p* < 0.05).

In addition, Figure 3 shows the changes in the concentrations of redox and nucleotide intermediates produced by the diet and the consumption of tomato juice. The effects were similar for reduced and oxidized glutathione (GSH and GSSG) as well as for NAD, NADH, and NADP, with lower values in the H groups than in the N groups. Consequently, the GSH/GSSG and NAD/NADH redox ratios were affected by the fatty diet and the steatosis, but amelioration was observed after the intake of tomato juice and the accumulation of lycopene in the liver of NL rats (Table 4).

## 4. Discussion

### 4.1. Steatosis Hallmarks

In developed countries, NAFLD is increasing due to the incidence of obesity, and it is currently more prevalent than alcohol-induced liver disease. Several studies have reported the beneficial effect of dietary carotenoids, particularly lycopene, for the prevention and treatment of steatosis [3,6,7,10].

Different authors have reported that high-fat and high-cholesterol diets caused steatosis, associated with adipose tissue inflammation, hypercholesterolemia and obesity [26]. In the present study, the experimental diet was used as a model to induce steatosis and obesity [27,28]; NAFLD was confirmed by the histological analysis of the liver and plasmatic dyslipidemia. Based on previous studies [6,10], we selected tomato juice as a natural source of lycopene, to identify the effect of dietary lycopene on the progression of steatosis and on the gene expression related to this pathology. Lycopene was absorbed and its digestion was facilitated by the consumption of the high-fat diet, since this permits the transport of this carotenoid through its introduction into lipid micelles and improves its absorption by enterocytes [6,29]. Consequently, lycopene (mainly all-*E* and 9-*Z*-lycopene) was detected in the liver of the animals of the NL and HL groups. Apo-lycopene was not detected, probably due to the preference for *cis* isomers of the enzyme carotenoid monooxygenase, which is repressed when β-oxidation does not function properly, as happens during steatosis [30].

Regarding the biochemical parameters, steatosis caused dyslipidemia in animals fed the H diet, whereas tomato juice consumption decreased the levels of total cholesterol in NL rats. The lipid-lowering effect of lycopene, found in healthy rats, has been reported in other studies in animals and humans [31], but it was not observed in animals with steatosis. However, in the HL group the final levels of cholesterol were increased, in comparison with the initial values, possibly due to its formation from VLDL, which indicates the transfer of lipids from the liver to the peripheral tissues, stimulated by lycopene [32]. Although animals of the H groups had ballooning hepatocytes, the histological examination showed that in the HA group the lipid vacuoles were larger than those of the HL group. This suggests that lycopene could have a preventive effect on NAFLD, reducing the accumulation of fat in hepatocytes and facilitating its transport to other tissues.

Another hallmark of steatosis is the oxidative stress associated with the increase in lipid peroxidation. This was confirmed by the higher levels of hepatic MDA in the H groups, relative to the N groups, indicating lipid oxidation in parallel with the advanced hepatic deterioration, verified by histological analyses [33,34]. In addition, the analysis of the urinary isoprostanes confirmed the status of oxidative stress in animals with induced steatosis. To evaluate the oxidative status, we also took into account the liver levels of GSH and GSSG, which were significantly lower in the H group, as well as the GSH/GSSG ratio, which also indicated great oxidative stress. Although the consumption of tomato juice did not yield a significant decline in the biomarkers of oxidation, in rats of the NL group there was a slight decrease in GSSG and, hence, a significant improvement in the GSH/GSSG ratio. This finding is in concordance with previous research in which we reported that lycopene favors the formation of glutathione, which restores the intracellular redox balance [6].

Taking into account that absorbed lycopene could prevent lipid accumulation in the liver and act as a dietary antioxidant, we evaluated its possible effect as a modulator of gene expression, using an array specific for fatty liver. In the scientific literature, NAFLD is associated with alterations in lipid metabolism; particularly, the synthesis of fatty acids and the esterification of triglycerides, modifying their oxidation in mitochondria and peroxisomes and the storage of lipids within the hepatocytes [35,36,37]. In a previous study, we reported that the consumption of tomato juice and the accumulation of lycopene modulated the expression of genes related to the β-oxidation of fatty acids [10]. In the present work we have investigated the effects on the genes involved in NAFLD.

### 4.2. Changes in Gene Expression Related to Fatty Liver

**Figure 4:**
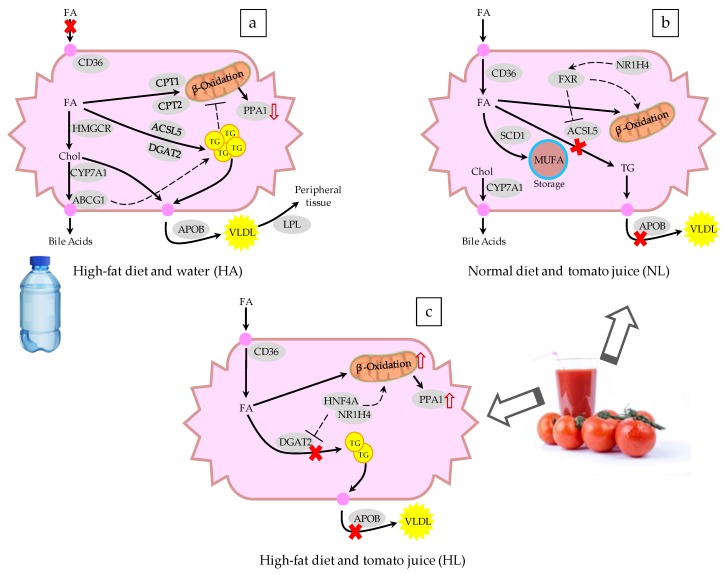
Schematic representation of the changes in the expression of genes involved in fatty liver disease after administering (**a**) a hypercholesterolemic and high-fat diet and water, (**b**) a normal diet and tomato juice, and (**c**) a hypercholesterolemic and high-fat diet and tomato juice to a murine model. FA: fatty acids, TG: triglycerides, VLDL: very low-density lipoprotein, Chol: cholesterol, MUFA: monounsaturated fatty acids. The continuous arrows indicate the flow of metabolites, the dashed arrows represent upregulation or downregulation, and the genes are represented as gray ovals. The genes are listed in Table 4.

The changes in the gene expression of the HA group were related to the steatosis (Figure 4a) [35,36,37]. A flow of cholesterol to the liver was shown, together with its subsequent excretion in the form of bile acids (*CYP7A1*) or its storage in vesicles (*ABCA1* and *ABCG1*), an activity that coincides with the occurrence of fatty vesicles in the rat liver described by Yvan-Charvet et al. [38]. Additionally, the activity of the *ACSL5* and *DGAT2* genes demonstrated greater synthesis of triglycerides [39,40]- which are excreted from the liver as the VLDL, releasing fatty acids in the tissues for their consumption and energy release [32]; this process is corroborated by the overexpression of *APOB* and *LPL* [41,42]. Therefore, the secretion of VLDL provides a mechanism for reduction of the intrahepatic TG; the secretion rate in subjects with NAFLD is not able to adequately compensate for the increased rate of accumulation of TG, so steatosis is maintained [43]. The overexpression of the *CPT1A* and *CPT2* transporters shows that there was an increase in mitochondrial β-oxidation in response to the higher accumulation of fat in the liver [44]. In addition, hepatocytes prevent the entry of more lipids by downregulation of *CD36*, closing the channel in the plasma membrane [42]. Another regulatory process involves an increase in the activity of the leptin receptor (*LEPR*) in adipose tissue, stimulating satiety to block food intake [45]. In contrast, the stimulation of cholesterol synthesis in HA animals is consistent with reports relating the expression of *HMGCR* to the severity of liver damage caused by steatosis [46]. Finally, the overexpression of *PPA1*, as found in HA rats, has due to its relationship with various metabolic reactions, been linked to disorders in lipid metabolism derived from NAFLD [10].

It is noteworthy that the intake of tomato juice influenced the entry of lipids into the hepatocytes through overexpression of *CD36* in both NL and HL rats, contrary to what was observed in HA rats (Figure 4b,c). Moussa et al. [47] reported that *CD36* functions as a trans-membrane transporter of carotenoids, mainly lycopene and lutein, which explains its activity when lycopene is present. In addition, the release of triglycerides was decreased by suppression of *APOB* and *LPL* [42], leading to accumulation of lipids in the hepatocytes, but the release of accumulated lycopene in HL rats was also avoided, favored by the activity of *FABP5*—which has been reported to be a transporter of long-chain lipid structures, such as lycopene and other carotenoids [48]. The mRNA of the liver X receptor (LXR), encoded by *NR1H2*, was overexpressed in NL rats. LXR has a homeostatic effect at the transcriptional level, which stimulates the synthesis of lipids via SREBF2 [49] and the excretion of bile acids, by activating *CYP7A1*, thereby reaching a balance in the content of hepatic cholesterol. The lipid accumulation produced by the membrane transporters and the lack of lipoprotein synthesis favor the expression of *ACOX1*, leading to the oxidation of lipids in peroxisomes and thus the recovery of the balance of hepatic lipids. Another modification of the lipid metabolism is the desaturation of fatty acids, due to the effect of *SCD1*, to give unsaturated fatty acids, thus decreasing their toxicity and avoiding their storage. Moreover, the synthesis of triglycerides is suppressed by a decrease in the activity of *ACSL5*, thus avoiding the accumulation of triglycerides, since the lipoprotein pathway is also inhibited [49]. The intake of tomato juice by healthy animals modulated the lipid metabolism and reduced the inflammation, due to the overexpression of *IL10*—the gene that encodes this anti-inflammatory cytokine. This effect could be related to the higher ratio of GSH/GSSG found in this group, since oxidative stress is implicated in inflammation and different metabolic disorders. Although tomato juice consumption did not have a great effect on the lipid metabolism of rats of the HL group, the upregulation of an inorganic pyrophosphatase (PPA1) suggests a high rate of mitochondrial oxidation of fatty acids, due to the metabolic flow of these compounds into the hepatocytes.

In the comparison of HL and HA rats, the mRNA of transcription factors *NR1H4* and *HNF4A* was overexpressed in the former. The *NR1H4* gene encodes the farnesoid X receptor (FXR), and its activation may have been involved in the reduction of liver steatosis and hyperlipidemia, through the suppression of de novo lipogenesis and the promotion of triglycerides oxidation and clearance. Moreover, FXR plays a crucial and beneficial role in glucose metabolism and in the regulation of bile acid homeostasis, and FXR agonists are promising for the treatment of NAFLD, dyslipidemia, and type 2 diabetes [50]. Recent studies have shown that lycopene and its metabolites resulting from the cleavage by the BCO1 and BCO2 enzymes could interact with the retinoic acid receptor (RAR). In the liver and intestine, FXR is expressed at high levels, and forms a heterodimer with RAR. The apolycopenoids resulting from the cleavage products of lycopene from tomato juice could be present in the liver at low concentrations and could act as ligands of the RAR [51,52]. When RAR heterodimerizes with other receptors, like FXR, it is involved in the regulation of its partner receptor’s pathways, such as bile acids, lipid, and glucose metabolism. The overexpression of FXR after the intake of tomato juice in animals with steatosis may be beneficial in the prevention and treatment of NAFLD, since it plays crucial roles in the mediation of multiple genes associated with lipid and glucose metabolism and the inflammation response [50]. The *HNF4A* gene encodes the hepatocyte nuclear factor 4α, and it is known to modulate regulatory elements in the promoters and enhancers of genes involved in cholesterol, fatty acid, and glucose metabolism [53,54]. This gene was upregulated in HL rats, in comparison with both NA and HA rats. In previous work, the mRNA expression of *HNF4A* was significantly decreased in human NASH samples, suggesting a contribution of *HNF4A* to NAFLD through regulation of the expression of the genes involved in the progression of NAFLD to hepatocellular carcinoma [55]. Here, the intake of tomato juice led to upregulation of the *NR1H4* and *HNF4A* genes, providing a protective effect against NAFLD. Ip et al. [56] reported that lycopene and its apo-derivatives can provide protection against hepatic steatosis by reducing the abundance of cholesterol and triglycerides in the liver, but plasmatic levels remain unchanged. They mentioned that these effects are due mainly to the activation of the *SIRT1* gene, which influences the inhibition of the expression of proteins involved in the synthesis of fatty acids, and promotes β-oxidation and the hydrolysis of triglycerides in fatty acids. Martín-Pozuelo et al. [10] observed that the intake of tomato juice modulated the expression of enzymes involved in the transport and oxidation of fatty acids. Similarly, we observed an upregulation of genes that encode hydrolytic protein (*LPL*) and fat transporter proteins (*CD36, FABP5*)—showing that for fatty acids both their transporters and their oxidative metabolism were active.

### 4.3. Changes in Liver Metabolites

Considering the results from the metabolomic study, the effect of the high-fat diet on the metabolism of amino acids and nucleotides deserves special attention; the contents of most of these compounds decreased, confirming the results obtained in previous studies with high-fat diets [6,57,58]. The amino acid arginine was one of the exceptions to the aforementioned behavior, its concentration increasing in the liver when the high-fat diet was supplied and its content in HA being higher than in HL rats. This could be due to the regulatory effect of arginine on the metabolism of lipids, since it modulates the expression and function of enzymes related to lipolysis and lipogenesis [59]. Tan et al. [60] reported that arginine stimulates lipolysis in adipocytes, promoting the oxidation of medium- and long-chain fatty acids and inhibiting lipogenesis in obese animals (with accumulation of adipose and insulin resistance). In addition, it appears to modulate the expression of genes related to lipids oxidation—such as *CPT1, PCG-1α*, and *MCD* in the liver- and also to repress the expression of *FAS* and *SCD1*. Jobgen et al. [59] showed that when arginine is present at relatively high concentrations it increases the oxidation of both glucose and oleic and palmitic acids, and stimulates the formation of triglycerides; despite this, arginine stimulates the incorporation of monounsaturated, rather than saturated, fatty acids. On the other hand, the intermediate amino acids of the redox process increased with the fat content of the diet, among them methionine and homo-cysteine. The homo-cysteine concentration was significantly increased in animals with steatosis, likely causing protein misfolding and stress in the endoplasmic reticulum [61]. This change might have been accompanied by a decrease in the content of cysteine, mainly due to the downregulation of cystathionine β-synthase and cystathionine γ-lyase, the enzymes responsible for the breakdown of homocysteine to cysteine, as described by Bravo and co-workers [62]. These are part of the methionine metabolic route, which is related to the pathway of transulfurization that is also linked to the biosynthesis of glutathione [62]. The above suggests that the limitation of glutathione synthesis is due to an imbalance in the hepatic homeostasis, producing an accumulation of glutathione precursors [6,62]. However, tomato juice consumption led to an increase in methionine levels, suggesting that the accumulation of lycopene in the liver could modulate the redox metabolism by increasing the level of this amino acid in order to raise the concentration of glutathione. Xie et al. [57] indicated that oxidative stress decreases the activity of antioxidant enzymes, including glutathione peroxidase and glutathione-S-transferase, and that this effect is exacerbated when NAFLD develops. In addition, the GSH/GSSG relationship showed significant differences in response to the H diet- similar to the findings of García-Cañaveras et al. [63], who mentioned a decrease in this ratio when steatosis was present, as a result of lipoperoxidation. Nevertheless, in the NL group, a significant increase (*p* < 0.05) in the redox ratio was observed due to the antioxidant activity of tomato juice, mainly lycopene, which acts to prevent lipid peroxidation. This effect has been reported [64,65] after the administration of pure lycopene and tomato extract, respectively, which act as antioxidants in the liver against the cytotoxicity of drugs. The NAD/NADH ratio was unaffected by changes in diet or intake of tomato juice; however, the liver values of NAD were higher with the standard (N) diet than with the fat diet (H). These results differ from those reported by Kim et al. [58], who observed a reduction in the NAD/NADH ratio in obese mice due to a decline in β-oxidation related to lipid accumulation. Nevertheless, in this study, despite the fact that an accumulation of lipids was found in the liver, no decrease in the lipid oxidative activity was observed, which would have resulted in an adequate balance in the energy metabolism.

## 5. Conclusions

We can conclude that the consumption of tomato juice improves the expression of genes encoding enzymes involved in lipid metabolism—thereby reducing the synthesis of fatty acids, triglycerides, and cholesterol, avoiding their accumulation, and modulating the progression of steatosis. In addition, the lycopene accumulated in the liver acts as a hepatic antioxidant, improving the redox balance in healthy rats. Although no changes in the biomarkers of oxidative stress were observed in animals with steatosis that had consumed tomato juice, the accumulation of lycopene appears to improve the levels of some amino acids related to antioxidant mechanisms and to modulate the expression of nuclear factors responsible for the regulation of lipid metabolism and cellular homeostasis, such as *FXR* and *HNF4A*. Therefore, based on the results obtained, tomatoes and tomato-derived products can be considered as part of a dietary strategy in the control and treatment of NAFLD, as natural and safe sources of lycopene. However, further studies should be conducted to determine the effect on the treatment of this pathology.

## Figures and Tables

**Table 1:** Food and drink intake, excreted feces and urine, lycopene intake, and apparent lycopene absorption of animals of the four experimental groups, in the 5-week intervention period.^1^

Parameters	NA	NL	HA	HL
Initial body weight (g)	428 ± 75 ^a^	432 ± 47 ^a^	375 ± 18 ^b^	406 ± 20 ^b^
Final body weight (g)	494 ± 65	496 ± 70	471 ± 20	489 ± 25
Body weight increase (%)	15.4	14.7	25.6	20.1
Food intake (g/day)	20 ± 2.6 ^a^	17.8 ± 1.8 ^b^	13.1 ±1.9 ^c^	12.0 ± 4.6 ^c^
Food intake (kcal/day) *	58	52	59	54
Tomato juice or water intake (mL/day)	25.8 ± 4.3 ^c^	63.6 ± 13.3 ^a^	44.3 ± 12.2 ^b^	78.8 ± 12.6 ^a^
Drinks intake (kcal/day) **	0	16	0	20
Total intake (kcal/day)	58	68	59	74
Excreted feces (g/day)	10.8 ± 1.2 ^a^	10.3 ± 0.9 ^a^	2.3 ± 1.4 ^b^	3.9 ± 1.3 ^b^
Excreted urine (mL/day)	12.2 ± 4.2 ^c^	19.5 ± 8.5 ^a,b^	15.4 ± 3.1 ^b,c^	33.5 ± 13.7 ^a^
Lycopene intake (mg/day)	nd	3.7 ± 0.8	Nd	4.6 ± 0.6
Excreted lycopene in feces (mg/day)	nd	1.57 ± 0.4	Nd	1.48 ± 0.2
Apparent lycopene absorption (%)	nd	55.7 ± 15.3	Nd	68.1 ± 11.4

^1^ Values are given as mean ± standard deviation (SD). ^a–c^ Different letters in the same row indicate significant differences for *p* < 0.05. * Data estimated as a function of the consumption of feed and its energetic value (N diet: 290 kcal/100 g and H diet: 450 kcal/100 g). ** Data estimated as a function of tomato juice intake and its energetic value (25 kcal/100 mL). NA (normal diet and water), NL (normal diet and tomato juice), HA (hypercholesterolemic diet and water) and HL (hypercholesterolemic diet and tomato juice).

**Table 2:** Hepatic content of lycopene metabolites, total lycopene, and its isomers, and liver weight in the four experimental groups, at the end of the 5-week intervention period ^1^.

Parameters	NA	NL	HA	HL
6-apo-lycopenal	nd	nd	Nd	nd
8-apo-lycopenal	nd	nd	Nd	nd
12-apo-lycopenal	nd	nd	Nd	nd
9-*cis* lycopene (µg/g)	nd	0.54 ± 0.10	Nd	0.81 ± 0.15
13-*cis* lycopene (µg/g)	nd	0.51 ± 0.26 *	Nd	0.25 ± 0.12
*Trans*-lycopene (µg/g)	nd	1.71 ± 0.30	Nd	3.49 ± 1.79 *
Total lycopene (µg/g)	nd	2.75 ± 0.33	Nd	4.55 ± 0.80 *
Liver weight (g)	11.91 ± 1 ^b^	12.82 ± 1.31 ^b^	22.74 ± 2.66 ^a^	24.12 ± 2.72 ^a^

^1^ Values are given as mean ± SD. * Indicates a statistically significant difference between the NL and HL groups according to a paired *t*-test (*p* < 0.05). ^a,b^ Different letters in the same row indicate significant differences for *p* < 0.05.

**Table 3:** Final plasma biochemical parameters, hepatic enzyme activities, and concentrations of urinary isoprostanes analyzed in the four experimental groups after the 5-week intervention period (NA: normal diet and water, NL: normal diet and tomato juice as drink, HA: hypercholesterolemic and high-fat diet and water, HL: hypercholesterolemic and high-fat diet and tomato juice).^1^

Parameters	NA	NL	HA	HL
Glucose (mg/dL)	136 ± 23	142 ± 40	158 ± 29	154 ± 33
Total cholesterol (mg/dL)	105 ± 24 ^b,c^	85 ± 20 ^c^	140 ± 42 ^a,b^	164 ± 12 ^a^
LDL-Cholesterol (mg/dL)	36 ± 9 ^b^	39 ± 9 ^b^	98 ± 28 ^a^	89 ± 9 ^a^
HDL-Cholesterol (mg/dL)	44 ± 9	33 ± 6	30 ± 4	34 ± 5
VLDL-Cholesterol (mg/dL)	13 ± 2 ^b^	11 ± 3 ^b^	20 ± 4 ^a^	24 ± 5 ^a^
Triglycerides (mg/dL)	66 ± 17 ^b^	63 ± 16 ^b^	98 ± 15 ^a^	110 ± 12 ^a^
ALT (U/L)	39 ± 5 ^b^	48 ± 15 ^b^	80 ± 2 ^a^	97 ± 29 ^a^
AST (U/L)	65 ± 9 ^b^	76 ± 14 ^b^	124 ± 20 ^a^	132 ± 24 ^a^
Urinary isoprostanes (pg/mL)	940 ± 86 ^b^	904 ± 55 ^b^	1303 ± 359 ^a^	1739 ± 200 ^a^

^1^ Values are given as mean ± SD. ^a–c^ Different letters in the same row indicate significant differences for *p* < 0.05.

**Table 4:** Concentration of malondialdehyde (MDA) and redox ratios in the liver of the rats of the four experimental groups (NA: normal diet and water, NL: normal diet and tomato juice as drink, HA: hypercholesterolemic and high-fat diet and water, and HL: hypercholesterolemic and high-fat diet and tomato juice). ^1.^

Parameters	NA	NL	HA	HL
MDA (nmol/mg of protein)	0.028 ± 0.003 ^b^	0.031 ± 0.003 ^b^	0.10± 0.02 ^a^	0.12 ± 0.02 ^a^
GSH/GSSG	0.188 ± 0.028 ^b^	0.252 ± 0.030 ^a^	0.114 ± 0.022 ^c^	0.136 ± 0.017 ^c^
NAD/NADH	2.3 ± 0.44	2.09 ± 0.49	2.76 ± 0.62	2.93 ± 0.89

^1^ Values are given as mean ± SD. ^a,b^ Different letters in the same row indicate significant differences for *p* < 0.05.

**Table 5:** Gene symbol, gene title, and relative fold change for the genes that showed an over or down expression value higher than 1.5 (*p* < 0.05) in the rat livers ^1^.

Symbol	Gene Name	NL–NA	HA–NA	HL–NA	HL–HA
***Fatty acid β-oxidation***					
*Cpt2*	Carnitine palmitoyltransferase 2	-	4.51	-	-
*Cpt1a*	Carnitine palmitoyltransferase 1a, liver	-	2.52	-	-
*Acox1*	Acyl-coenzyme A oxidase 1, palmitoyl	1.90	-	-	-
***Cholesterol transport and metabolism***					
*Abca1*	ATP-binding cassette, subfamily A (ABC1), member 1	-	2.39	-	-
*Hmgcr*	3-hydroxy-3-methylglutaryl-coenzyme A reductase	-	1.52	-	-
*Cd36*	CD36 molecule (thrombospondin receptor)	5.84	−3.28	4.23	-
*Pparg*	Peroxisome proliferator-activated receptor gamma	-	1.64	-	-
*Abcg1*	ATP-binding cassette, subfamily G, member 1	-	2.43	-	-
*Cyp7a1*	Cytochrome P450, family 7, subfamily a, polypeptide 1	1.52	1.51	-	-
*Lepr*	Leptin receptor	-	2.43	-	-
*Nr1h2*	Nuclear receptor subfamily 1, group H, member 2	1.66	−1.68	-	-
*Nr1h4*	Nuclear receptor subfamily 1, group H, member 4	-	-	-	1.80
*Srebf2*	Sterol regulatory element binding transcription factor 2	2.27	-	-	-
*Apob*	Apolipoprotein B	−6.43	6.80	−4.66	-
***Other lipid transport and metabolism***					
*Scd1*	Stearoyl-coenzyme A desaturase 1	2.09	-	-	-
*Lpl*	Lipoprotein lipase	−4.05	6.22	−3.33	-
*Acsl5*	Acyl-coa synthetase long-chain family member 5	−1.64	1.90	-	-
*Dgat2*	Diacylglycerol O-acyltransferase homolog 2 (mouse)	-	1.51	-	-
*Ppa1*	Pyrophosphatase (inorganic) 1	-	−2.20	3.57	-
*Fabp5*	Fatty acid binding protein 5, epidermal	-	-	2.99	-
*Hnf4a*	Hepatocyte nuclear factor 4, alpha	-	-	1.76	1.79
*IL10*	Interleukin 10	2.83	-	-	-

^1^ The fold change for each gene in groups HA, HL, and NL was calculated taking as reference a value of 1 for the group NA. For comparison, HA vs. HL reference values of 1 have been given to group HA.
